# Assessing Virulence Factor Genes in Pig-Derived *Escherichia coli* from the Region of Vojvodina Treated with Postbiotic Substance and Herbal Essential Oils

**DOI:** 10.3390/pathogens15020215

**Published:** 2026-02-13

**Authors:** Andrea Lauková, Jana Ščerbová, Valentína Focková, Igor Stojanov, Monika Pogány Simonová, Jasna Prodanov-Radulović

**Affiliations:** 1 Centre of Biosciences of the Slovak Academy of Sciences, Institute of Animal Physiology, Šoltésovej 4-6, 040 01 Košice, Slovakia; scerbova@saske.sk (J.Š.); fockova@gmail.com (V.F.); simonova@saske.sk (M.P.S.); 2 Scientific Veterinary Institute Novi Sad, Rumenački put 20, 21000 Novi Sad, Serbia; igor@niv.ns.ac.rs (I.S.); jasna@niv.ns.ac.rs (J.P.-R.)

**Keywords:** virulence factor, gene, *Escherichia coli*, postbiotic, essential oil, treatment

## Abstract

Antibiotic-resistant, biofilm-forming *Escherichia coli* may constitute a reservoir of antibiotic resistance and other determinants that can be transmitted to pathogenic bacteria for animals and humans. Therefore, it is crucial to reduce the incidence of these types of *E. coli*. The aim of this study was to determine whether essential oils from oregano, thyme, sage, and coriander, as well as the postbiotic substance PS412, can inhibit virulence factor genes possessing pig-derived *E. coli.* It aimed to find a new tool for the prevention and/or elimination of virulent *E. coli*. Altogether, 16 pig-derived *E. coli* from a pig farm in the region of Vojvodina (Serbia) were taxonomically identified using MALDI-TOF mass spectrometry; 14 strains (87.5%) with secure genus identification/probable species identification and 2 with highly probable genus identification. The *fimA* gene was detected in 62.5% of *E. coli* strains, and the *crl* gene in 87.5% of the strains. Ec3419/2 contained five analyzed genes. Five *E. coli* were found to form biofilm, as indicated by their growth on Congo red agar. The strains were mostly multi-resistant to antibiotics. Each *E. coli* strain produced the damaging enzyme, such as β-glucuronidase and/or α-chymotrypsin. However, they were susceptible to herbal essential oils (HEOs) with average inhibitory zones from 15 to 27 mm in diameter. They were also (6) susceptible to the PS412 (activity to 6400 AU/mL). The results contribute to the practical effectiveness of postbiotic substances, HEOs, and their combination as a novel approach to combating the virulence factors of *E. coli*. This insight also contributes to the strategy behind the One Health Concept.

## 1. Introduction

In general, the prevalence of antibiotic-resistant bacteria is a growing concern due to the possible transmission of resistant bacteria or their resistance genes between animals and humans via direct and/or indirect contact [1]. Antibiotic-resistant *Escherichia coli* may constitute a reservoir of antibiotic resistance determinants. The determinants can be spread to those bacteria that are pathogenic for animals and humans [1]. The biofilm-forming ability and so biofilm development also represent a problem [2]. Although Gram-negative species of *Escherichia coli* have been recognized as a component of the healthy intestinal microbiota [3], they can differ in their capacity to persist in the normal colonic microbiota. Resident strains may colonize an individual for months or years, while transient strains are found only once or on a few occasions closely spaced in time in an individual’s intestine [3]. On the other side, diseases caused by some *E. coli* species are one of the most important diseases, and they represent a public health risk [4,5]. *E. coli* synthesize a variety of adhesins that allow them to colonize, persist, and/or cause disease. There are filaments called fimbriae that allow bacteria to adhere to a variety of cells [6]. This enhances *E. coli* biofilm formation [7]. The curli regulatory gene (*crl*) is responsible for the production of curli fibers in *E. coli* [6]. The *HlyA* (α-hemolysin) is a toxin responsible for the strain’s pathogenicity [8]. Strains containing the *eae* gene (outer membrane protein-intimin) frequently cause diarrhea, as do those containing the *espA* gene (superficial protein) as well. Therefore, it is very important to control and/or reduce the growth of these types of *E. coli* strains.

Postbiotic substances (not homogenously purified) and bacteriocins (homogenously purified), which are proteinaceous antimicrobial substances with an inhibitory effect against related or unrelated bacteria, represent one of these approaches [9]. Their inhibitory effect (e.g., Enterocin-like, and/or Enterocin-Ent substances) was also presented in several of our studies. Lauková et al. [10] reported the effective treatment of contaminant strains of *Enterococcus faecalis* (from raw goat milk) due to postbiotic active substances produced by autochthonous lactococci. Pogány Simonová et al. [11] reported a reduction in fecal coliforms (*p* < 0.001) and pseudomonads (*p* < 0.05) in broiler rabbits after the application of postbiotics, Ent M and Durancin ED26E/7. Postbiotic substance PS412, indicated previously as Ent 412, is a thermo-stable antimicrobial proteinaceous substance (that is not homogenously purified up to now) produced by the horse strain *Enterococcus faecium* EF412 [12]. This strain is susceptible to antibiotics and exhibits adhesion ability to human and canine mucus [13]. Ent 412 (PS412) was applied in horses with the following benefits noted: coliforms were significantly inhibited (*p* < 0.001) after 3 weeks of Ent 412 application; phagocytic activity was increased (69.0 ± 3.99 at day 21, when at the start of experiment it was 67.89 ± 3.66); and biochemical parameters in the blood were not negatively influenced (Lauková et al., unpublished data).

Additionally, herbal essential oils are recognized for their known antimicrobial effect [14,15,16]. Herbal essential oils and their components, as products of the secondary metabolism of plants, have been known to possess antimicrobial and antioxidant properties for a long time, used in preservation, food, and the pharmaceutical industry [14]. For example, carvacrol (2-methyl-5-(1-methylethyl)-phenol) is a major component of the oregano essential oil. It was reported to inhibit *E. coli*, *Listeria monocytogenes*, *Salmonella Enterica*, and *Campylobacter jejuni* [17]. Thymol (5-methyl-2-(1-methylethyl)-phenol) has been commercially used for a hundred years [15]. Its essential oil has been shown to exhibit a range of biological activities, e.g., inhibition of aflatoxin production. Its antimicrobial activity was also documented against *E. coli*, Listeriae, and Campylobacters [15]. Sage (*Salvia officinalis* L.; family Labiatae) essential oil in combination with Ent M increased the mucus production quantity in the duodenum and jejunum (*p* < 0.001 and *p* < 0.01) in broiler rabbits after their 3-week application in comparison with control rabbits [18]. Moreover, *Coriandrum sativum* L. (Apiaceae) essential oil has been reported to inhibit the growth of Gram-negative species, such as *E. coli*, *Yersinia enterocolitica*, *Enterobacter* spp., and *Klebsiella pneumoniae* [16]. Therefore, carvacrol, thyme, and oregano-containing oils have attracted research attention for further application.

Given this information, the aim of this study was to analyze whether four selected essential oils (oregano, thyme, sage, and coriander) and the postbiotic substance PS412 can inhibit virulence factor genes possessing pig-derived *E. coli* to find another new tool for the prevention and/or elimination of those type strains of *E. coli*.

## 2. Materials and Methods

### 2.1. Taxonomical Identification of Isolates

Here, isolates were selected from 26 rectal swabs and 9 samples from animals that had died. Sampling was conducted on a commercial, industrial-type pig farm with production organized from farrowing to finishing in the Vojvodina region of Serbia, as part of a collaboration with our colleagues [19]. In general, the production categories on the farm included sows, gilts, boars, suckling piglets, weaned piglets, and pigs in fattening. The farm has been continually facing diarrhoeal problems. Sampling was conducted in accordance with the rules for animal handling, as accepted by farmers and veterinarians, and in accordance with appropriate veterinary administration in Serbia. Colleagues in Serbia immersed the swabs in sterile saline solution (pH 7.5) and transported them to the laboratory in cooling boxes on the same day [19]. The samples were inoculated onto Mac Conkey agar (Biokar Diagnostics, Allonne, France), XLD agar (Biokar, Allonne, France), and Trypticase soy agar (TSA, Biokar, Allonne, France). TSA was enriched with 5% defibrinated sheep blood [19]. Phenotypic identification of grown colonies was performed by colleagues in Serbia using biochemical tests (oxidase and catalase tests, indole, methyl red, urea, and citrate). After supplying the identified strains in our laboratory (Laboratory of Animal Microbiology, Department of Animal Physiology, Centre of Biosciences of the Slovak Academy of Sciences in Košice, Slovakia), the strains were pre-inoculated using Trypticase soy broth (Difco, Sparks, MD, USA) and Mac Conkey agar (Difco, Sparks, MD, USA) and analyzed by MALDI-TOF mass spectrometry (Matrix-Assisted-Laser Desorption/Ionization Time of Flight Mass Spectrometry; Bruker Daltonics, San Jose, CA, USA).

The MALDI-TOF analysis was performed as previously described by Lauková et al. [20,21]. This method uses protein detection “fingerprints” (Bruker Daltonics, Biotyper 2.2) [20,21,22,23], generated with a Microflex MALDI-TOF mass spectrometer. Briefly, a single colony of each isolate from Trypticase soy agar (Difco, Sparks, MD, USA) with 5% defibrinated sheep blood was mixed with a matrix (α-cyano-hydroxycinnamic acid and trifluoroacetic acid). The suspension was spotted onto a MALDI plate and ionized with a nitrogen laser (wavelength of 337 nm; frequency of 20 Hz). The identification score was determined using the MALDI Biotyper 3.0 (Bruker Daltonics, Billerica, MA, USA) identification database. Strains were classified as highly probable species identification (score range 2.300–3.000), secure genus identification/probable species identification (2.000–2.299), and highly probable genus identification (1.700–1.999). Strains in the identification database served as positive controls. Identical colonies with the same score were excluded. Finally, 16 strains were used for the next analyses. The Microbank system (Pro-Lab Diagnostic, Richmond, BC, Canada) was used to store identified strains.

### 2.2. Virulence Factor Genes’ Detection of Pig-Derived Escherichia coli

The following virulence factor genes were screened: *fimA* (fimbriae), *crl* (curli regulatory gene), *hlyA* (hemolysin), *eaeA* (outer membrane protein-intimin), and *espA* (superficial protein). The primers used are summarized in Table 1. Single PCR was performed using the Thermocycler Techgene KRD (Techne, London, UK) and the protocol for *fimA* gene detection reported previously by Nowrouzian et al. [3]: an initial denaturation step at 94 °C for 3 min, followed by 30 cycles of DNA denaturation at 94 °C for 1 min, primer annealing at 63 °C for 1 min, a primer extension at 72 °C (1 min) and a final extension step at 72 °C for 7 min. For the *hlyA* gene, the PCR conditions included an initial denaturation at 94 °C (2 min), followed by 30 cycles at 94 °C (1 min), an annealing at 58 °C for 1 min, primer extension at 72 °C (1 min), and a final extension at 72 °C (7 min) [24]. The *eaeA* gene was amplified using a PCR protocol that included an initial denaturation at 94 °C (1 min), 30 cycles at 94 °C for 30 s, an annealing at 55 °C for 30 s, an extension at 72 °C (1 min), and a final extension at 72 °C for 10 min [25]. The conditions used for the *espA* gene were 94 °C for 5 min, 40 cycles at 94 °C for 30 s, annealing at 50 °C (1 min), extension at 72 °C (1 min), and a final extension at 72 °C (10 min) [26]. For the *crl* gene, initial denaturation was performed at 94 °C for 2 s, followed by 30 cycles at 55 °C for 3 s, an annealing step at 55 °C, and an extension at 72 °C for 15 s [6]. Amplifications were carried out in a single tube with a volume of 25 µL, using Taq polymerase (0.2 µL, Promega, Madison, WI, USA), 10 × buffer (2.5 µL), MgCl_2_ (1.6 µL), primers (0.2 µL each, Lambda Life, Bratislava, Slovakia), deoxynucleotide triphosphate (dNTP, Promega, Madison, WI, USA), and water (17.4 µL). The components were mixed. Specific gene products were analyzed by electrophoresis on a 1.5% agarose gel (Sigma-Aldrich, Steinheim am Albuch, Germany) in 1× TAE (Tris-acetate-EDTA, Merck, Germany) gel at 70 V. The products were visualized with GelRed (Biotium Inc., Hayward, CA, USA) under UV light. The 100 bp ladder (Promega, Madison, WI, USA) served as a molecular weight standard to determine the molecular weight of the products. Positive controls were *E. coli* Ec A/Zn/2021 and EcK212Tr (our strains).

### 2.3. Biofilm-Forming Ability Testing

Two methods were used for this testing. The qualitative method was based on biofilm-forming ability using Congo red agar [27]. The medium consisted of TSY broth (Difco, MI, USA, 37 g/L) with 30 g/L pure agar, 36 g/L sucrose, and Congo red dye (0.8 g/L, Merck, Germany). *E. coli* were inoculated onto Congo red agar and incubated at 37 °C for 24 h. The biofilm-forming ability of *E. coli* was demonstrated by black colonies with a dry crystalline consistency. The non-biofilm-forming strains remained pink. The color was also checked after 48 h and 72 h, with plates placed at room temperature [28].

The quantitative microtiter plate assay [29] was also used for biofilm testing. An individual colony of the *E. coli* strain grown on Trypticase soy agar for 18 h (TSA, Difco, Sparks, MD, USA) was transferred into 5 mL of Ringer solution (pH 7.0; Merck, Darmstadt, Germany) to reach 1.0 × 10^8^ CFU/L. A 100 µL aliquot of this suspension was transferred into 10 mL of Trypticase soy broth/infusion (BHI). A 200 µL aliquot of this dilution was inoculated into microtiter plate wells (Greiner ELISA 12 Well Strips, 350 µL (Greiner Elisa 12 Well Strips flat bottom, Frickenhausen GmbH, Frickenhausen, Germany) as previously reported by Lauková et al. [30]. After overnight incubation at 37 °C, the biofilm formed in the microtiter wells was washed twice with 200 µL of deionized water and dried at 25 °C for half an hour. The next step was to stain the remaining bacteria for 30 min at 25 °C with 200 µL of 0.1% (*w*/*v*) crystal violet in deionized water. The plates were dried again (half an hour at room temperature). The dye bound to the adhered biofilm was extracted using 200 µL of 95% ethanol and stirred. A 150 µL aliquot was again transferred to a microplate well, and absorbance at A_570_ (Synergy TM4 MALDI mode Microplate reader (Biotek, Seattle, WA, USA) was used for measurement. Two independent analyses with 12 replicates were performed for each *E. coli* strain. A sterile BHI was included in each test as a negative control. The positive control was *Streptococcus equi* subsp. *zooepidemicus* CCM 7316 (kindly provided by Dr. Eva Styková from the University of Veterinary Medicine and Pharmacy in Košice, Slovakia). The biofilm-forming ability of the strain *E. coli* was evaluated as reported by Chaieb et al. [29]. The following classification was used: highly positive (A570 ≥ 1), low-grade positive (0.1 ≤ A570 < 1.0), or negative (A570 < 1.0).

### 2.4. Antibiotic Disc Diffusion Test and Enzyme Activity Detection

To test the susceptibility of *E. coli* to antibiotics, the agar disc diffusion method [30] was applied. The antibiotics (13) were supplied by Oxoid Ltd. (Basingstoke, UK) as follows: clindamycin (DA—2 µg), penicillin (P—10 IU), ampicillin (Amp—10 µg), gentamicin (CM—10 µg), erythromycin (E—15 µg), azithromycin (AZM—15 µg), amikacin (AK—30 µg), chloramphenicol (C—30 µg), tetracycline (T—30 µg), mezocillin (Mez—75 µg), ticarcillin (Tic—75 µg), carbenicillin (Car—100 µg), and piperacillin (Prl—100 µg). Briefly, broth cultures (100 µL) of the tested strains in Trypticase soy broth (Difco, Sparks, MD, USA) were spread onto Mueller–Hinton agar (Bio-Rad, Bratislava, Slovakia) and onto Trypticase soy agar (Difco, Sparks, MD, USA). After antibiotic discs were applied to the agar surface, the agar plates were incubated at 37 °C for 18 h. After incubation, the inhibitory zones were measured and evaluated in accordance with the guidelines of the Clinical and Laboratory Standards Institute [30]. Testing was performed in duplicate. *E. coli* ATCC 25922 served as a positive control.

The API ZYM panel (Bio Mérieux, Marcy l’Etoile, France) was used for enzyme detection according to the manufacturer’s instructions, as previously reported by Lauková et al. [31]. Briefly, 65 µL of McFarland standard inoculum was transferred into each well of the tested panel plate. After incubation of the panel plate at 37 °C for 4 h in the incubator, reagents Zym A and Zym B were added to each well. Enzyme activity was evaluated based on color intensity values (0–5) and their corresponding values in nanomoles (nmoL). These values were assigned to each reaction according to the color chart supplied with the kit. The enzymes tested in the panel were as follows: alkaline phosphatase, esterase (C4), esterase lipase (C8), lipase (C14), leucine arylamidase, valine arylamidase, cystine arylamidase, trypsin, α-chymotrypsin, acid phosphatase, Naftol-AS-BI-phosphohydrolase, α-galactosidase, β-galactosidase, β-glucuronidase, α-glucosidase, β-glucosidae, N-acetyl-β-glucosaminidase, α-mannosidase, and α-fucosidase.

### 2.5. Susceptibility to Herbal Essential Oils of E. coli

The essential oils used were obtained from Calendula a.s. Nová Ľubovňa (Slovakia). In testing, the following were included: Salviae aetheroleum from sage (*Salvia officinalis* L.; family Labiatae) contained cineol (15.0 ± 10.0.5%), thujon (24.0 ± 1.0%), and borneol (18.0 ± 1.0). Oregano aetheroleum from *Origanum vulgare* L. (Lamiaceae) contained 55.0 ± 3.0% of carvacrol. Coriandri aetheroleum from *Coriandrum sativum* L. (Apiaceae) contained 53.0 ± 2.0% of the main component linalool. Thymi aetheroeum from thyme (*Thymus vulgaris* L., Lamiaceae) contained 40.0 ± 3.0% of p-cymene, and 32.0 ± 2.0% of thymol. The components of these oils were measured using gas chromatography. The agar spot test [32] was used to assess the effect of essential oils against *E. coli* strains. For this purpose, *E. coli* were grown in TSY (18 h). A volume of 4 mL of soft agar (*w*/*v* 0.7%) was inoculated with 200 µL of each tested strain, and TSY (*w*/*v*, 1.5%) was overlaid with this mixture. Then, 10 µL of each essential oil (EO) was dropped onto the plate surface. The plates were incubated at 37 °C, and after 4 h, the first check was performed. The second check was performed after overnight incubation. The susceptibility of *E. coli* was evaluated as the average size of the inhibitory zone (in mm) ± SD. *Escherichia coli* ATCC 25922 was used as a positive control. Testing was performed in duplicate.

### 2.6. Postbiotic Substance 412 Preparation and Susceptibility to PS 412 of Pig-Derived E. coli

The PS 412 was prepared according to the procedure of Mareková et al. [33]. MRS broth (200 mL, de Man-Rogosa-Sharpe, pH 7, Merck, Germany) was inoculated with a 14–18 h culture of *Enterococcus faecium* EF412 (0.1% inoculum) at 37 °C. The culture was centrifuged (10,000× *g*) for 30 min. The supernatant was adjusted to pH 5.5 and precipitated with ammonium sulfate (40% saturation) in a plastic jar by stirring at 4 °C for 1 h or longer, depending on the precipitation process. A second centrifugation (10,000× *g*) was then performed for 30 min. The pellets from the centrifuged tubes were resuspended in a minimal volume of 10 mM phosphate buffer (pH 6.5). The inhibitory activity of the precipitate (postbiotic substance) was tested against *E. avium* EA5 (the principal indicator, our strain) using an agar spot test [32] and expressed as AU/mL after dilution in phosphate buffer. The activity reached 51,200 AU/mL. *E. coli* strains were then treated with PS as previously indicated, and the analysis was performed in duplicate.

## 3. Results

### 3.1. Strains Taxonomy Using MALDI-TOF Mass Spectrometry

Identified *E. coli* strains with an evaluated score are summarized in Table 2. Sixteen strains were taxonomically assigned and confirmed as *Escherichia coli.* Fourteen strains (87.5%) achieved a score of 2.000–2.299, indicating secure genus identification or probable species identification. Strains *E. coli* Ec3419/4 and Ec3298/4 were evaluated and yielded a score of 1.700–1.999, indicating highly probable genus identification (Table 2). The strain Ec3419/1 achieved the highest score (2.238).

### 3.2. Virulence Factor Genes, Biofilm-Forming Ability, Enzyme Activity, and Antibiotic Profile of Escherichia coli

In total, 6 of 16 *E. coli* strains lacked the *fimA* gene; however, the *fimA* gene was present in 10 strains (62.5%, Table 2). The *crl* gene was detected in 14 strains (87.5%); only 2 strains (Ec7676/4 and Ec3477/1) were *crl* gene-negative. The *E. coli* strains tested were mostly negative for the *hlyA*, *eae,* and *espA* genes (11 strains in each, as shown in Table 2). These genes were detected in five strains. *E. coli* Ec3419/2 possessed all five analyzed genes. The strain Ec3298/1 possessed four genes (the *espA* gene was not detected). Three genes were most commonly found in the tested strains. In the strain Ec3419/4, only the *crl* gene was present. The strains Ec7676/4 and Ec3477/1 were devoid of the virulence factor gene.

Using Congo red agar to test biofilm-forming ability in *E. coli*, this ability was observed in five strains (31.2%, Table 2); a dubious reaction was noted in three strains, and eleven strains did not show biofilm-forming ability using Congo red agar. In dubious reactions, negative production was confirmed using the quantitative plate assay. Values less than 0.1 were measured (Table 2) in almost all tested strains, even in those with a positive reaction on Congo red agar. In three negative strains tested using Congo red agar, no biofilm-forming ability was also confirmed by the plate agar assay (Table 2). In general, measurements ranged from 0.006 to 0.050.

Each of the *E. coli* tested was found to have enzymatic activity with varying values (Table 3). The values of esterase (C4), lipase (C14), leucin-arylamidase, valin-arylamidase, cystin-arylamidase, trypsin, α-glucosidase, β-glucosidase, α-mannosidase, and α-fucos idase reached 5 nmoL in each tested *E. coli*. Higher values (10–20 nmoL) were measured for alkaline phosphatase. Regarding esterase-lipase and α-galactosidase, most strains reached a value of 5 nmoL, except for Ec3152 and Ec3419/6, which had a value of 10 nmoL, as did Ec3298/4. The enzyme α-chymotrypsin was also produced by the tested *E. coli* in an amount of 5 nmoL, except Ec3419/6 (10 nmoL). The values for acidic phosphatase and Naftol-AS-BI-phosphohydrolase were almost the same; the values 10 and 20 nmoL dominated, except in the strain Ec7612/4 for acidic phosphatase (30 nmoL), and for the strains Ec3298/3, 3298/4 in the case of Naftol-AS-BI-phosphohydrolase (30 nmoL). The volume of 5 nmoL was found for α-galactosidase, except Ec3298/4 (10 nmoL). For β-galactosidase, the values 5 nmoL up to 10 nmoL were noted. In the case of N-acetyl-β-glucosaminidase, only Ec7612/4 reached 30 nmoL; the other strains produced 5 nmoL of this enzyme.

Results of the following enzymes are included in Table 3; 1: alkaline phosphatase, 3: esterase lipase (C8), 9: α-chymotrypsin, 10: acidic phosphatase, 11: Naftol-AS-BI-phospho-hydrolase, 12: α-galactosidase, 13: β-galactosidase, 17: N-acetyl-β-glucosaminidase. In the following enzymes, 2: esterase (C4), 4-lipase (C14), 5: leucine arylamidase, 6: valine-arylamidase, 7: cystine-arylamidase, 8: trypsin, 14: β-glucuronidase, 15: α-glucosidase, 16: β-glucosidase, 18: α mannosidase, and 19: α-fucosidase, the reached values were 5 nmoL.

The *E. coli* strains were resistant to clindamycin and penicillin. Conversely, they were all susceptible to amikacin (AK30) with the inhibitory zones up to 13 mm. In total, 11 strains out of 16 (Table 3, 68.8%) were resistant to chloramphenicol (C30), and 5 strains were susceptible to this antibiotic (Table 3). Moreover, 13 strains (81.3%) were resistant to tetracycline (T30), and 12 strains (75%) were resistant to ampicillin (Amp10). Surprisingly, 8 strains were gentamicin (CM10)-resistant, and 8 strains were CM10-susceptible. Fifteen *E. coli* (15) were resistant to erythromycin (E15, 93.8%). *E. coli* were mostly resistant to azithromycin (AZM15), at 11 strains (68.8%), as well as to mezlocillin (Mez75), at 10 strains of *E. coli* (62.5%). The same number of *E. coli* strains (10, 62.5%) were resistant to ticarcillin (Tic75, Table 4). The tested *E. coli* (16) were also mostly resistant to Car100 (carbenicillin)—15 out of 16 strains (93.8%). The opposite was observed with piperacillin (Prl100): almost 12 strains were susceptible, while 6 were resistant. In general, the strains were mostly resistant to the antibiotics.

### 3.3. Susceptibility of E. coli to Herbal Essential Oils and Postbiotic Substances

All tested *E. coli* strains were susceptible to oregano, thyme, sage, and coriander (Table 5). The average inhibitory zone after *E. coli* treatment with oregano was 27.0 ± 0.0 mm. *E. coli* Ec3419/1 was the least susceptible to oregano, with an inhibitory zone of 16 mm. The most susceptible was *E. coli* Ec3276, with an inhibitory zone of 36 mm. Treatment with oregano produced the broadest inhibitory zones (Table 5). The second essential oil to which *E. coli* strains were susceptible was thyme. The average inhibitory zone size was 18 mm. The strain Ec3419/2 could be considered resistant based on a small inhibitory zone (5 mm). The broadest zone, 25 mm, was observed in Ec3419/3. In the case of sage and coriander, the average inhibitory zones reached 15 mm. The strains Ec3298/1, Ec3298/2, Ec3298/3, and Ec3298/4 were the most susceptible to essential oils and to PS 412 (Table 5), with inhibitory activity ranging from 100 to 6400 AU/mL. Ec3298/4 was the most susceptible to PS 412 (6400 AU/mL). Ten strains were not inhibited by PS 412; however, their growth was inhibited by herbal essential oils.

## 4. Discussion

The first important step in working with selected microbiota is their identification. In this study, after phenotyping, MALDI-TOF MS was used as the main identification method. It is an accurate method for laboratory use because its simple approach is designed for protein detection. It represents a reliable technology for the precise identification of cultured microbiota [20]. This method appears to be a reliable identification tool for the species *E. coli*, achieving a high score. It was also successfully applied to the identification of fecal *E. coli* from ostriches [34]. As is known, the species *E. coli* belongs to the family Enterobacteriaceae, the order Enterobacterales, the class Gammaproteobacteria, and the phylum Pseudomonata (Proteobacteria). As previously mentioned, *E. coli* is a common commensal bacterial species found in animals and humans that can become a troublesome pathogen, causing disease [35].

Regarding virulence factors, the curli gene *crl* is most commonly present in avian *E. coli* [6]. The eae protein (intimin) contributes to damage of cells’ microvilli. It is a key virulence factor associated with diarrheal disease [35]. Intimin is critical for bacterial ability to adhere to intestinal cells. Strains containing the *eae* gene are responsible for causing diarrhea [35]. In our strains, five were found to possess the *eaeA* gene. The *fimA* gene is a crucial virulence factor in *E. coli* for adhesion to host cells and tissue colonization. It is an adhesin that helps bacteria attach to host tissues, allowing them to colonize and establish infections [3]. The *espA* gene encodes a protein essential for activating epithelial signal transduction, establishing intimate contact, and forming attaching and effacing lesions, processes central to pathogenesis [26]. The *hlyA* gene is a member of the RTX toxin family. It is believed that the persistence of α-hemolytic *E. coli* strains in the host may contribute to the emergence of intestinal and/or extra-intestinal infections [36]. In this study, the *crl* gene was detected most frequently.

The *E. coli* studied here exhibited multidrug resistance, which may pose a problem for pig farming in Serbia and globally. Multidrug resistance was defined as acquired non-susceptibility to at least one agent in three or more antimicrobial categories [37]. The transfer of microbial resistance genes poses a significant challenge to the permanent control of antimicrobial resistance. As previously reported by Stojanov et al. [19], 100% resistance to penicillin was observed in *E. coli* from piglets at the grower stage; we also found the same results in *E. coli* from pigs in the Vojvodina region. Roderová et al. [35] reported 97% resistance to ampicillin and 96% resistance to Prl among *E. coli* from hospitalized patients. This contrasts with our 100% susceptibility to Prl. Gentamicin is an older aminoglycoside. Half of our tested *E. coli* strains were resistant, and the other half (8) were susceptible to gentamicin. Similarly, Kerluku et al. [38] observed gentamicin resistance in *E. coli* with low resistance levels in cattle and sheep. Similarly, as noted by Kerluku et al. [38], moderate resistance to azithromycin was also observed in our strains. Stojanov et al. [19] confirmed the development of multidrug resistance in pigs on farms in the Vojvodina region (Serbia). This resistance manifested as multidrug resistance to a group of antibiotics, including penicillins, synthetic penicillins, aminoglycosides, fluoroquinolones, and tetracyclines. Therefore, finding alternatives for use in this context is encouraging for farmers.

The tested *E. coli* strains produce several harmful enzymes. The production of enzymes such as α-chymotrypsin, β-glucuronidase, and alkaline phosphatase contributes to the strain’s pathogenic character. Alkaline phosphatase serves as a diagnostic marker for hepatitis. The enzyme β-glucuronidase, when derived from microbial sources, can play a role in disease related to estrogen metabolism [39].

Naturally occurring substances were found to be effective in inhibiting or reducing *E. coli* infections [14,15]. Among these naturally occurring substances is the lantibiotic bacteriocin nisin, which was effectively used in combination with cinnamaldehyde and EDTA to control swine-origin *E. coli* [40]. In our case, the postbiotic substance, Enterocin-like PS412, inhibited the growth of 6 of 16 strains. It is interesting that in experimental in vivo applications using various animals, most frequently broiler rabbits, poultry, and/or horses, a reduction in *E. coli* was noted with a beneficial influence observed for this and other Enterocins, Enterocin-like substances, and/or postbiotics [41,42]. The beneficial preventive use of Ent7420 enhances the growth and immunity of rabbits and also provides protection against infection caused by methicillin-resistant staphylococci. Underlining the effectiveness of postbiotic substances, Petrová et al. [43] reported new findings. The direct effect of Enterocins (Ent M and Durancin ED26E/7) on *Trichinella spiralis* fecundity was documented in an in vitro test. There, Durancin-like showed a reduction effect (40–60%). The reduction activity in *T. spiralis* infection induced by Ent M was also noted [43]. Moreover, Al Atya et al. [44] reported inhibition of swine-derived *E. coli* in both planktonic and biofilm cultures using LAB-bacteriocins (nisin + Enterocin DD14) in combination with colistin. The author even outlines the advantages of antibiotic-LAB bacteriocin combinations and suggests that they merit further development as potential novel treatments for *E. coli* infections. Here, the tested *E. coli* did not form biofilm when tested by the quantitative method. Discrepancies between qualitative and quantitative biofilm results can be attributed to differences in the aspects measured, such as the cell count versus matrix mass and metabolic activity. They can also be influenced by environmental factors, cell density, and matrix composition [45]. The postbiotic 412 use approach was supported by the use of HEO. In some experiments [15], the authors also indicated that dietary administration of a combination of thyme and oregano essential oils in appropriate concentrations can reduce the production of pro-inflammatory cytokines, attenuate the degree of colonic tissue injury, and ameliorate colitis in mice. In our case, the strains Ec3298/1, Ec3298/2, Ec3298/3, and Ec3298/4, which possess virulence factor genes, were the most susceptible to essential oils and to PS412. The mechanism of the postbiotic and HEOs involves synergistic effects. The postbiotic substance provides stable support for gut health and modulates immunity, while HEOs exert direct antimicrobial activity, disrupting cell membranes and metabolism of pathogens. The combination enhances overall microbial balance, improving nutrient absorption and strengthening gut integrity more effectively than either component alone. The postbiotic supports the host and HEOs more frequently than the pathogen cell directly [46]. However, to our knowledge, the postbiotic also directly inhibits pathogen cells, which could indicate greater synergistic potential. Therefore, this study contributes to identifying a possible strategy for protecting and/or eliminating non-requested microbiota, such as virulence factor genes that *E. coli* possesses. Although it is necessary to continue this testing, it also provides insight into how to address multidrug-resistant *E. coli* on farms in the Vojvodina region.

## 5. Conclusions

Pig-derived *Escherichia coli* strains with virulence factor genes, multidrug resistance, and the ability to produce damaging enzymes were susceptible to essential herbal oils (oregano, sage, thyme, and coriander). Moreover, 6 of 16 strains were susceptible to the postbiotic (Enterocin-like) substance PS412. These results contribute to the development of a new tool, such as a postbiotic substance in combination with herbal essential oils, to achieve the final elimination of virulence factor genes in *E. coli*. Although this study presents a limited number of *E. coli* strains, it serves as a means to demonstrate to farmers not only in the Vojvodina region how to treat diarrheal problems in pigs. To maintain healthy animals, it is essential to maintain human and environmental health; resistant strains pollute the environment. These aspects fit within the One Health concept.

## Figures and Tables

**Table 1:** Oligonucleotide primer pairs to detect virulence factor genes in *Escherichia coli* strains.

Gene	Oligonucleotide Primer Pairs	Size of PCR Product (bp)	Reference
*crl (curli regulatory gene)*, *M571*, *M570*	F:5′-TTTCGATTGTCTGGCTGTATG-3′	250	[6]
	R:5′-CTTCAGATTCAGCGTCGTC-3′		
*eaeA (outer membrane protein-intimin*, *non-fimbrial adhesin)*, *eaeA 1*, *eaeA 2*	F:5′-CTGAACGGCGATTACGCGAA-3′	917	[25]
	R:5′-CCAGACGATACGATCCAG-3′		
*espA (protein secreted by enteropathogenic E. coli)*, *espA1*, *espA2*	F:5′-GCGAGTACTTCGACATC-3′	579	[26]
	R:5′-TTATTTACCAAGGGATAT-3′		
*hlyA (α-hemolysin)*, *hly1*, *hly2*	F:5′-GTCTGCAAAGCAATCCGCTGCAAATAAA-3′	1177	[24]
	R:5′-CTGTGTCCACGAGTTGGTTGATTAG-3′		
*fimA (fimbrial adhesin)*, *fimA 1*, *fimA 2*	F:5′-ACGTTTCTGTGGCTCGACGCATCT-3′ R:5′-ACGTCCCTGAACCTGGGTAGGTTA-3′	721	[3]

**Table 2:** The MALDI-TOF evaluating score, virulence factor genes, and biofilm-forming ability of *E. coli*.

Strain	MALDI-TOF	*fimA*	*crl*	*hlyA*	*eaeA*	*espA*	Congo 72 h	Biofilm ± SD
Ec3298/1	2.067	+	+	+	+	−	−	0.011 ± 0.009
Ec3298/2	2.127	−	+	−	+	+	±	0.006 ± 0.002
Ec3298/3	2.209	−	+	−	+	+	−	0.005 ± 0.003
Ec3298/4	1.897	+	+	−	−	−	+	0.050 ± 0.01
Ec3098/1	2.081	+	+	+	−	−	+	0.007 ± 0.003
Ec3098/2	2.265	−	+	−	+	+	−	-
Ec3152	2.135	+	+	+	−	−	−	-
Ec3276	2.222	+	+	+	−	−	−	0.027 ± 0.02
Ec3419/1	2.238	+	+	−	−	−	−	-
Ec3419/2	2.197	+	+	+	+	+	±	0.013 ± 0.004
Ec3419/3	1.988	+	+	−	−	−	±	0.025 ± 0.004
Ec3419/4	2.216	−	+	−	−	−	−	0.013 ± 0.003
Ec3419/6	2.213	+	+	−	−	−	+	0.018 ± 0.005
Ec3717/1	2.016	+	+	−	−	+	+	0.015 ± 0.006
Ec7676/4	2.060	−	−	−	−	−	+	0.048 ± 0.012
Ec3477/1	2.204	−	−	−	−	−	−	0.039 ± 0.014

Virulence factor genes: *fimA* (fimbriae), *crl* (curli regulatory factor), *hlyA* (hemolysin), *eaeA* (outer membrane protein-intimin), *espA* (superficial protein). Growth of *E. coli* strains on Congo red agar (biofilm formation); + indicates positive reaction; − indicates a negative reaction; ± indicates a dubious reaction.

**Table 3:** Enzyme production by the tested *E. coli* expressed in nmoL.

Strain	1	3	9	10	11	12	13	17
Ec3298/1	10	5	5	20	20	5	5	5
Ec3298/2	10	5	5	20	20	5	10	5
Ec3298/3	10	5	5	20	30	5	10	5
Ec3298/4	20	5	5	20	30	10	10	5
Ec3098/1	10	5	5	20	20	5	5	5
Ec3098/2	10	5	5	10	20	5	5	5
Ec3152	20	10	5	20	20	5	5	5
Ec3276	20	5	5	20	20	5	20	5
Ec3419/1	20	5	5	20	20	5	5	5
Ec3419/2	20	5	5	10	20	5	10	5
Ec3419/3	10	5	5	10	20	5	10	5
Ec3419/4	10	5	5	10	10	5	5	5
Ec3419/6	20	10	10	20	20	5	5	5
Ec7162/2	20	5	5	10	20	5	5	5
Ec7612/4	20	5	5	30	30	5	5	30
Ec3477/1	20	5	5	20	20	5	5	5

**Table 4:** Antibiotic profile of the tested *E. coli*.

Strain	C30	T30	Amp10	CM10	E15	AZM15	AK30	Mez75	Tic75	Car100	Prl100
Ec3298/1	R	R	R	+14	R	R	+12	R	R	R	+10
Ec3298/2	+22	R	R	+12	R	+11	+10	R	R	R	+11
Ec3298/3	+20	R	R	+11	R	+10	+10	R	R	R	+10
Ec3298/4	R	R	R	R	R	+10	+11	R	R	R	R
Ec3098/1	+20	R	R	+11	R	R	+12	R	R	R	+13
Ec3098/2	R	R	R	R	R	R	+12	R	R	R	R
Ec3152	+20	R	R	+11	R	+10	+10	R	R	R	+10
Ec3276	R	+12	R	+11	+13	+10	+11	+10	+10	R	+10
Ec3419/1	R	R	+15	R	R	+9	+10	+15	+15	R	+9
Ec3419/2	R	R	R	+10	R	R	+10	R	R	R	+12
Ec3419/3	+23	+17	R	+11	R	+10	+10	R	R	R	R
Ec3419/4	R	R	+15	R	R	R	+10	+13	+14	R	+16
Ec3419/6	R	R	R	R	R	+10	+13	+15	+15	+10	+20
Ec7162/2	R	+8	+17	R	R	+8	+10	+10	+11	R	+17
Ec7612/4	R	R	+12	R	R	R	+10	+10	+10	R	+10
Ec3477/1	R	R	R	R	R	R	+10	R	R	R	R

C30, chloramphenicol (30 µg); T30, tetracycline (30 µg); Amp10, ampicillin (10 µg); CM10, gentamicin (10 IU); E15, erythromycin (15 µg); AZM15, azithromycin (15 µg); AK30, amikacin (30 µg), Mez75, mezlocillin (75 µg) Tic75, ticarcillin (75 µg); Car100, carbenicillin (100 µg); Prl100, piperacillin (100 µg); R, resistant; + (no.), inhibitory zone size in mm. The *E. coli* strains were resistant to clindamycin and penicillin.

**Table 5:** Susceptibility to postbiotic substance PS (Ent-like) 412 expressed in arbitrary units per mL and to herbal essential oils in pig-derived *E. coli* (inhibitory zone diameter—mm), with ± SD.

Strain	Oregano	Thyme	Sage	Coriander	PS (Ent) 412
Ec3298/1	35.0 ± 0.0	21.0 ± 0.0	15.0 ± 0.0	21.0 ± 0.0	800.0 ± 0.0
Ec3298/2	22.0 ± 0.0	27.0 ± 0.0	24.0 ± 0.0	31.0 ± 0.0	100.0 ± 0.0
Ec3298/3	19.0 ± 0.0	10.0 ± 0.0	20.0 ± 0.0	1.0 ± 0.00	1600 ± 0.0
Ec3298/4	30.0 ± 0.0	21.0 ± 0.0	19.0 ± 0.0	19.0 ± 0.0	6400 ± 0.0
Ec3098/1	30.0 ± 0.0	21.0 ± 0.0	21.0 ± 0.0	15.0 ± 0.0	-
Ec3098/2	30.0 ± 0.0	15.0 ± 0.0	13.0 ± 0.0	10.0 ± 0.0	-
Ec3152	30.0 ± 0.0	24.0 ± 0.0	13.0 ± 0.0	19.0 ± 0.0	-
Ec3276	36.0 ± 0.0	14.0 ± 0.0	10.0 ± 0.0	15.0 ± 0.0	-
Ec3419/1	16. 0± 0.0	20.0 ± 0.0	9.0 ± 0.0	10.0 ± 0.0	3200 ± 0.0
Ec3419/2	30.0 ± 0.0	5.0 ± 0.0	10.0 ± 0.0	12.0 ± 0.0	-
Ec3419/3	23.0 ± 0.0	25.0 ± 0.0	10.0 ± 0.0	7.0 ± 0.0	1600 ± 0.0
Ec3419/4	27.0 ± 0.0	20.0 ± 0.0	15.0 ± 0.0	10.0 ± 0.0	-
Ec3419/6	20.0 ± 0.0	20.0 ± 0.0	10.0 ± 0.0	12.0 ± 0.0	-
Ec7162/2	20.0 ± 0.0	15.0 ± 0.0	10.0 ± 0.0	10.0 ± 0.0	-
Ec7612/4	21.0 ± 0.0	14.0 ± 0.0	10.0 ± 0.0	10.0 ± 0.0	-
Ec3477/1	35.0 ± 0.0	13.0 ± 0.0	10.0 ± 0.0	10.0 ± 0.0	-

## Data Availability

Data will be provided by the corresponding author upon request.
